# Lymph node ratio as a predictor for minor salivary gland cancer in head and neck

**DOI:** 10.1186/s12885-021-08877-3

**Published:** 2021-11-06

**Authors:** Hidenori Suzuki, Eiichi Sasaki, Gaku Takano, Seiya Goto, Daisuke Nishikawa, Shintaro Beppu, Hoshino Terada, Michi Sawabe, Nobuhiro Hanai

**Affiliations:** 1 Department of Head and Neck Surgery, Aichi Cancer Center Hospital, Nagoya, Aichi 464-8681 Japan; 2 Department of Pathology and Molecular Diagnostics, Aichi Cancer Center Hospital, Nagoya, Japan; 3 Department of Otolaryngology, Nagoya City University Graduate School of Medical Sciences and Medical School, Nagoya, Japan

**Keywords:** Lymph node ratio, Log odds of positive lymph nodes, Overall survival, Minor salivary gland carcinoma

## Abstract

**Background:**

We investigate whether pathological continuous variables of lymph nodes were related with survival results of carcinomas of minor salivary gland carcinoma in head and neck.

**Methods:**

Forty-four cases with minor salivary gland carcinoma who underwent both primary resection and neck dissection were retrospectively enrolled. The pathological continuous variables were evaluated by the number of positive lymph nodes, lymph node ratio, and log odds of positive lymph nodes. Receiver operating curve analysis was used for the cut-off values of the carcinoma-specific death. Log-rank test and Cox’s proportional hazards model were used for uni−/multi-variate survival analyses adjusting for pathological stage, respectively.

**Results:**

Lymph node ratio = 0.05 as well as log odds of positive lymph nodes = − 2.73 predicted the carcinoma-specific death. Both lymph node ratio and log odds of positive lymph nodes were significantly related with survival outcomes by the univariate analysis. Lymph node ratio ≥ 0.05 was associated with shorter disease-specific (hazard ratio = 7.90, 95% confidence interval = 1.54–57.1), disease-free (hazard ratio = 4.15, 95% confidence interval = 1.48–11.2) and overall (hazard ratio = 4.84, 95% confidence interval = 1.05–24.8) survival in the multivariate analysis.

**Conclusion:**

A higher lymph node ratio of minor salivary gland carcinoma is a predictor of shorter survival results.

## Background

Lymph node on pathological examination is investigated as useful predictors of survival results in several types of cancer [1, 2]. Representative continuous variables of lymph nodes was the number of positive lymph nodes after neck dissection surgery [2]. Both lymph node ratio (LNR) and log odds of positive lymph nodes (LODDS) as pathological continuous variables, which were regulated by nodal staging, surgery, and sampling, were applied regardless of various types for neck dissection [2–4]. For the absence of positive lymph nodes described, LNR or LODDS represent the same value = 0 or avoids singularities, respectively [1].

Minor salivary gland carcinoma (MiSGC) is a rare neoplasma in head and neck, accounting for 0.16 to 0.4% of new cases per 100, 000 population [5]. Mucoepidermoid carcinoma (MEC) and adenoid cystic carcinoma are histologically reported two most common classifications, and the definitive treatment for MiSGC is surgery with or without postoperative radiation [6]. Although the pathological predictors for MiSGC of the head and neck were indicated in a recent review article [7], other predictors must be determined as this is a rare malignancy.

Therefore, we aimed to investigate whether LNR and LODDS in patients with MiSGC were significantly correlated with survival outcomes.

## Methods

### Patient selection

This retrospective study according to the Declaration of Helsinki was performed at the Department of Head and Neck Surgery in our institution, and approved by our institutional review board (receipt number 2019–1-427). Forty-seven patients with MiSGC in head and neck who were newly diagnosed without distant metastasis underwent neck dissection and primary tumor resection between July 2003 and June 2019. Among them, three patients who received preoperative chemotherapy were excluded. Thus, 44 patients who received lymph node biopsies for pathological diagnosis of lymph node and informed consent for examinations and interventions were enrolled. The extent of elective neck dissection was mostly submental, submandibular, upper jugular, and middle jugular lymph nodes. The extent of therapeutic neck dissection was mainly submental, submandibular, upper jugular, middle jugular, lower jugular, spinal accessory, and supraclavicular lymph nodes.

### Clinicopathological parameters

The oral cavity (*n* = 28), sinonasal tract, (*n* = 8), and pharynx (n = 8) were the primary sites of MiSGC. The pathological restaging of MiSGC in each primary site was conducted according to the 8th Edition of the American Joint Committee on Cancer staging manual [8]. Details of the interventions, pathological examinations, LNR, pathological TNM restaging based on the 8th edition of the Union for International Cancer Control (UICC), and follow-up were described previously [3, 9]. Histological grade, perineural invasion, vascular invasion, and worst pattern of invasion from primary tumor were pathologically assessed by an experienced pathologist. Smoking history and American Society of Anesthesiologists-Physical Status (ASA-PS) were reviewed as patient demographic factor. The clinicopathological parameters (age, sex, primary site, pathological T and N category, pathological stage, extranodal extension, positive surgical margin, type of neck dissection, postoperative intervention, and histological classification, histological grade, perineurial invasion, vascular invasion, worst pattern of invasion, smoking history, smoking history, and ASA-PS) are presented in Table 1.

**Table 1:** Parameters in 44 patients with MiSGC in the head and neck

Parameter		Number
Age (year)	Mean ± standard deviation	59.5 ± 11.6
Sex	Male/female	18/26
Pathological T classification	T1/T2/T3/T4	7/9/11/17
Pathological N classification	N0/N1/N2a/N2b/N2c/N3a/N3b	26/4/1/7/3/0/3
Pathological stage	I/II/III/IVA/IVB	3/6/11/21/3
Primary site	Oral/pharynx/sinonasal tract	28/8/8
Positive surgical margin	Presence/absence	10/34
Extranodal extension	Presence/absence	4/40
Type of neck dissection	Unilateral/bilateral	36/8
Postoperative treatment	Radiation/chemoradiation/Absence	11/1/32
Histological classification	MEC	15
	Adenoid cystic carcinoma	13
	Adenocarcinoma, not otherwise specified	7
	Carcinoma ex pleomorphic adenoma	6
	1Undifferentiated carcinoma	2
	Acinic cell carcinoma	1
Histological grade	Low/intermediate/high	5/25/14
Perineural invasion	Presence/absence	15/29
Vascular invasion	Presence/absence	19/25
Worst pattern of invasion	1–3/4/5	12/26/6
Smoking history	Presence/absence	18/26
ASA-PS	1/2	17/27

### Pathological continuous variables

The number of positive lymph nodes, LNR, and LODDS were evaluated as continuous variables of pathological lymph node. LNR was calculated as the number of positive lymph nodes/the total number of resected lymph nodes [3, 9]. LODDS were computed as follows: log [(number of positive lymph nodes + 0.5)/(total number of resected lymph nodes-number of positive lymph nodes + 0.5)], as described by Safi et al. [2].

### Statistical analysis

Pathological continuous variables were evaluated using linear regression test. The Kaplan-Meier curves was estimated by the survival time from surgery to last date of contact or an aim event. Death from MiSGC (MiSGC-specific survival), local recurrence (local recurrence-free survival [LRFS]), regional recurrence (regional recurrence-free survival [RRFS]), distant metastasis (distant metastasis-free survival [DMFS]), recurrence or metastasis (disease-free survival [DFS]), and death (overall survival [OS]) were the aim events. Applying previous method by conducting a receiver operating curve (ROC) analysis [9], various cut-off values for pathological continuous variables were tested in the death due to MiSGC. All patients were categorized into two groups: those with LNR < .05 vs. ≥.05 and those with LODDS <-2.73 vs. ≥ − 2.73. The deviations in clinicopathological parameters or survival results between the two groups were compared by Fisher’s exact test or the log-rank test, respectively. Pathological N classification (pN0-pN3) was also analyzed as potential rick factor in addition to the number of positive lymph nodes by ROC analysis, Fisher’s exact test, log-rank test, multivariate survival analysis. Multivariate analyses of MiSGC-specific survival, DFS, and OS were conducted by five Cox proportional hazards regression with hazard ratio (HR) as well as 95% confidence interval (95% CI). Model 1 was adjusted for LNR (≥.05/<.05) and pathological stage (IVB/I-IVA). Model 2 was adjusted with LODDS (≥ − 2.73/<− 2.73) and pathological stage (IVB/I-IVA). Model 3 was adjusted for pathological category (N1–3/N0) and pathological stage (IVB/I-IVA). Model 4 was adjusted for LNR (≥.05/<.05), pathological stage (IVB/I-IVA) and vascular invasion (Presence/Absence). Model 5 was adjusted with LODDS (≥ − 2.73/<− 2.73), pathological stage (IVB/I-IVA) and vascular invasion (Presence/Absence). Given positive lymph nodes/number of lymph nodes/extranodal extension is a central component of overall the 8th edition staging, it is possible that factors that involve positive lymph nodes (LNR or LODDS) and pathologic staging are highly correlated and collinearity is present in the multivariate analysis. We perform interaction test between pathological stage (IVB/I-IVA) and either of LNR (≥0.05/< 0.05)/LODDS (≥ − 2.73/<− 2.73) by the multivariable Cox model. A *p-*value of < 0.05 was considered as significant. Statistical analyses were conducted by the JMP version 9 (SAS: Cary, NC, USA).

## Results

### Linear regression analysis

**Fig. 1:**
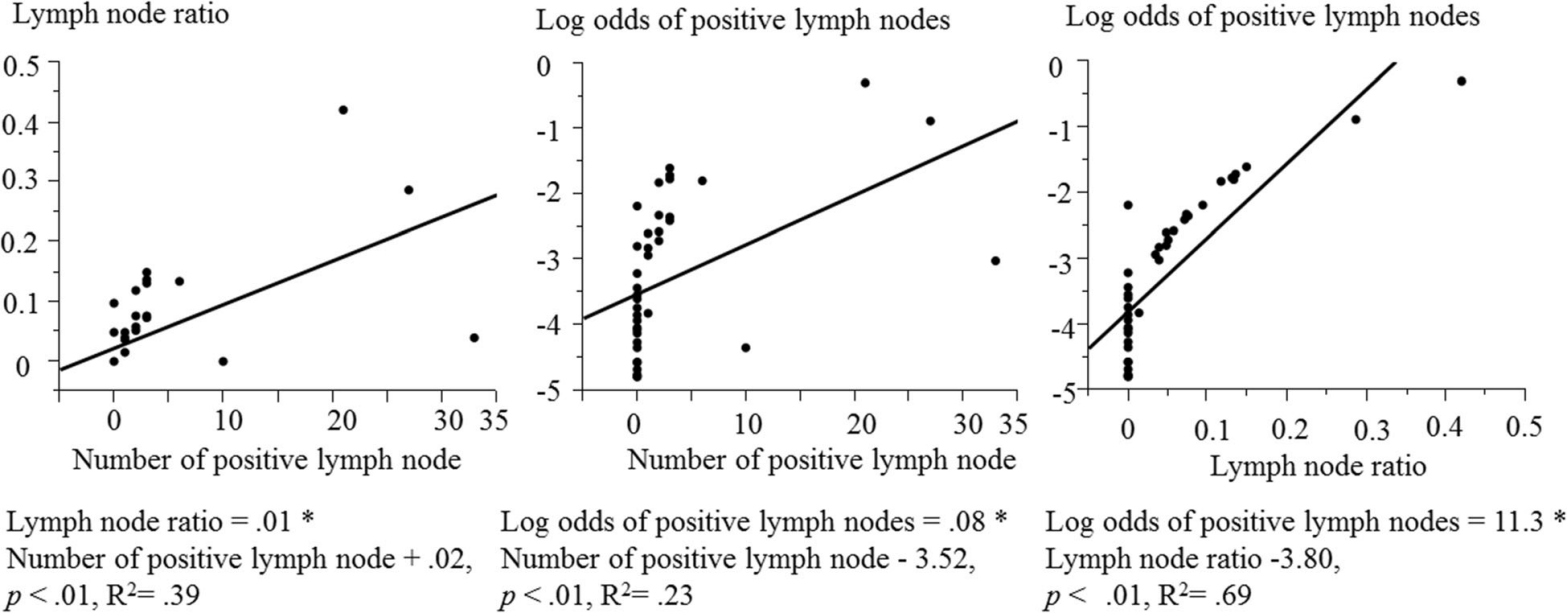
Linear regression curves for 44 carcinomas in minor salivary gland

The linear regression analyses are exhibited in Fig. 1. Both LNR (*p* < .01, R^2^ = .39) and LODDS (*p* < .01, R^2^ = .23) was linearly the number of positive lymph nodes. LNR was linearly LODDS (*p* < .01, R^2^ = .69). The mean ± standard deviation of the number of positive lymph nodes, LNR, LODDS, and the total number of resected lymph nodes, were 2.75 ± 7.02, 0.04 ± 0.08, and − 3.31 ± 1.12, and 36.6 ± 21.8, respectively. The median (interquartile range) for the number of positive lymph nodes, LNR, LODDS, and the total number resected lymph nodes were 0 (2–0), 0 (0.06–0), − 3.58 (− 2.47--4.13), and 28 (49–21), respectively.

### Survival results

The mean ± standard deviation continuance of follow-up at long last in the study was 6.38 ± 3.95 years for all patients, 7.17 ± 3.78 years for the 35 survivors, 2.40 ± 1.70 years for the 8 patients who died because of MiSGC, and 3.33 ± 3.21 years for the 9 patients who died. The median (interquartile range) follow-up at long last in the study was 5.78 (9.19–3.12) years for all patients, 6.34 (9.28–4.50) years for the 35 survivors, 1.51(4.40–1.40) years for the 8 patients who died because of MiSGC, and 1.54 (4.91–1.40) years for the 9 patients who died. Three (6.82%, vs all) cases developed local recurrence, five (11.4%, vs all) developed regional recurrence, and nine (36.4%, vs all) had distant metastasis. The 5 year MiSGC-specific survival, LRFS, RRFS, DMFS, DFS, and OS rates were 79.6, 91.6, 88.0, 62.9, 56.9, and 79.6%, respectively.

### ROC analysis

**Fig. 2:**
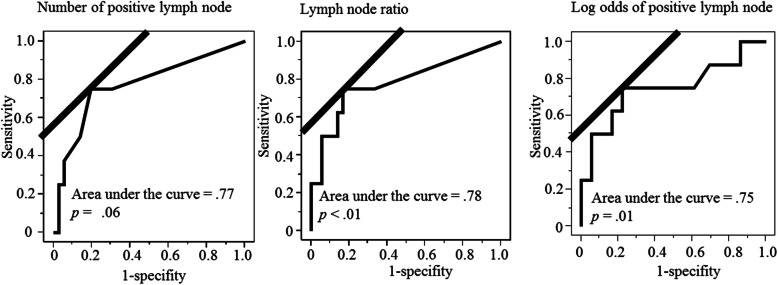
Receiver operating curves in 44 carcinomas of minor salivary gland

The ROC analyses for death from MiSGC are shown in Fig. 2. The topper cut-off values to continuous pathological variables were LNR = 0.05, LODDS = − 2.73, and the number of positive lymph nodes = 2. Death from MiSGC were significantly predicted by both LNR = 0.05 (*p* < .01, area under the curve = .78) and LODDS = − 2.73 (*p* = .01, area under the curve = .75). However, there were no significant association between the number of positive lymph nodes = 2 and death from MiSGC (*p* = .06, area under the curve = .77). The cut-off value to the pathological N classification was pathological N1 classification, and death from MiSGC was significantly predicted pathological N1 (*p* = .02), area under the curve = .74).

### Log-rank test for the two LNR groups

**Fig. 3:**
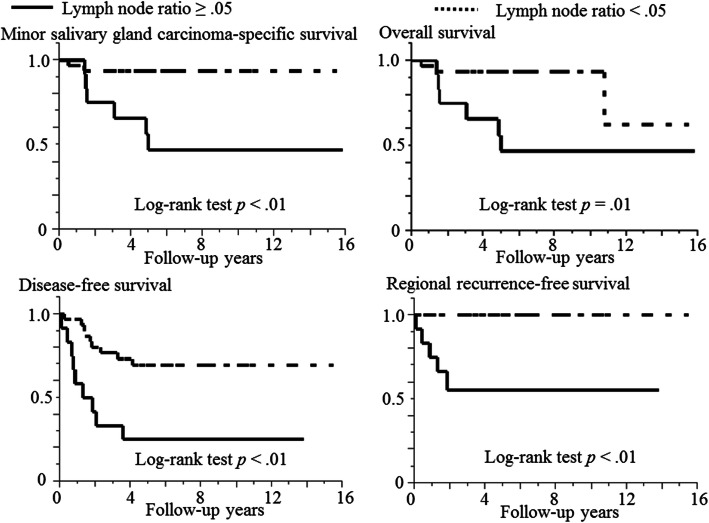
Kaplan-Meier curves in 44 patients with minor salivary gland carcinoma divided into two groups of lymph node ratio

The representative curves of the Kaplan-Meier method for the two LNR groups are presented in Fig. 3. Cases with LNR ≥ .05 were significantly associated with shorter MiSGC-specific survival (*p* < .01), OS (*p* = .01), DFS (*p* < .01), and RRFS (*p* < .01) compared with those with LNR < .05. Conversely, any significant deviation was not showed in the two LNR groups for LRFS (*p* = .07) or DMFS (*p* = .07).

### Log-rank test of the two LODDS groups

**Fig. 4:**
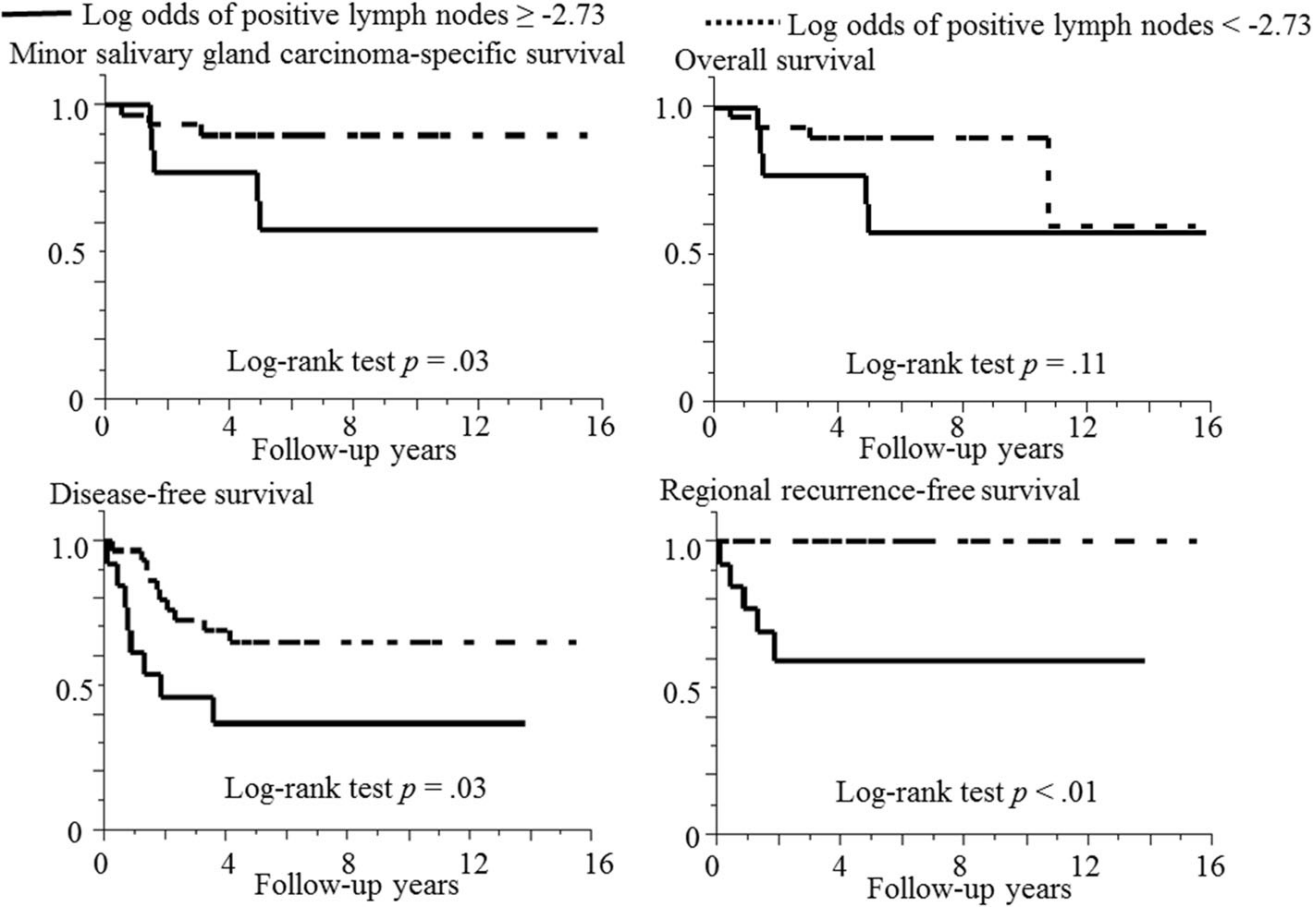
Kaplan-Meier curves of 44 cases of minor salivary gland carcinoma separated into two groups for log odds of positive lymph nodes

The representative Kaplan-Meier curves of the two LODDS groups are shown (Fig. 4). Cases with LODDS ≥ − 2.73 were significantly related with shorter MiSGC-specific survival (*p* < .01), DFS (*p* = .03), and RRFS *p* < .01) than those with LODDS < − 2.73. Conversely, no significant difference was found in the two LODDS groups for LRFS (*p* = .10), DMFS (*p* = .16), or OS (*p* = .11).

### Log-rank test of the two pathological N classification groups

Cases with pathological N 1–3 classification were closely related to poorer MiSGC-specific survival (*p* = .03), DFS (*p* = .01), RRFS (*p* < .01), and DMFS (*p* < .01) than cases with pathological N0 classification. Conversely, no significant relation was observed in the two pathological N classification groups for OS (*p* = .08) and LRFS (*p* = .24).

### Fisher’s test of the two groups

The relationship in terms of clinicopathological parameters between the two groups is shown in Table 2. Pathological N1-N3b (*p* < .01) and stage IVB (*p* = .02) were more frequently in LNR ≥ .05 compared with LNR < .05. LODDS ≥ − 2.73 were frequently female (*p* = .02), had pathological N1-N3b (*p* < .01) and stage IVB (*p* = .02) in comparison to LODDS < − 2.73.

**Table 2:** Association between clinicopathological parameters and the two groups (LNR, LODDS and pN category) evaluated using Fisher’s test

Parameter		LNR			LODDS			pN category		
		≥ .05 (*n* = 12)	<.05 (*n* = 32)	*p* value	≥ − 2.73 (*n* = 13)	<−2.73 (*n* = 31)	*p* value	N1–3 (*n* = 18)	N0 (*n* =26)	*p* value
Age	≥ 60	7	15		7	15		10	12	
	< 60	5	17	.74	6	16	1.00	8	14	.76
Sex	Male	7	11		9	9		9	9	
	Female	5	21	.18	4	22	.02	9	17	.36
Pathological T category	T1-T3	5	22		7	20		9	18	
	T4	7	10	.16	6	11	.52	9	8	.23
Pathological N category	N0	0	26		1	25				
	N1-N3b	12	6	<.01	12	6	<.01			
Pathological stage	I-IVA	9	32		10	31		15	26	
	IVB	3	0	.02	3	0	.02	3	0	.06
Primary site	Oral	7	21		7	21		10	18	
	Others	5	11	.73	6	10	.50	8	8	.52
Positive surgical margin	Presence	5	5		4	6		6	4	
	Absence	7	27	.11	9	25	.45	12	22	.27
Extranodal extension	Presence	3	1		3	1		4	0	
	Absence	9	31	.06	10	30	.07	14	26	.02
Type of neck dissection	Unilateral	9	27		10	26		13	23	
	Bilateral	3	5	.66	3	5	.68	5	3	.24
Postoperative treatment	Presence	5	7		4	8		6	6	
	Absence	7	25	.26	9	23	.73	12	20	.51
Histological classification	MEC	4	11		4	11		6	9	
	Others	8	21	1.00	9	20	1.00	12	17	1.00
Histological	Low	0	5		0	5		1	4	
grade	Others	12	27	.30	13	26	.30	17	22	.63
Perineural	Presence	4	11		3	12		5	10	
invasion	Absence	8	21	1.00	10	19	.49	13	16	.53
Vascular	Presence	10	9		9	10		12	7	
invasion	Absence	2	23	<.01	4	21	.04	6	19	.01
Worst pattern	1–3	2	10		2	10		3	9	
of invasion	4–5	10	22	.46	11	21	.46	15	17	.30
Smoking	Presence	6	12		7	11		9	9	
history	Absence	6	20	.51	6	20	.32	9	17	.36
ASA-PS	1	5	12		5	12		8	9	
	2	7	20	1.00	8	19	1.00	10	17	.54

Presence of vascular invasion compared to absence of vascular invasion were frequently in LNR ≥ .05 (*p* < .01) and LODDS ≥ − 2.73 (*p* = .04). Pathological N1–3 category in comparison to pathological N0 category were frequently observed in the presence of both extranodal extension (*p* = .02) and vascular invasion (*p* = .01).

### Five models of Cox’s proportional hazards regression

The multivariate analyses are shown in Table 3. In Model 1, LNR ≥ .05 were significantly poorer MiSGC-specific survival (*p* = .01, HR: 7.90, 95% CI: 1.54–57.1), DFS (*p* = .01, HR: 4.15, 95% CI: 1.48–11.2), and OS (*p* = .04, HR: 4.84, 95% CI: 1.05–24.8), than LNR < .05. In Model 2, no significant associations were found between LODDS (≥ − 2.73/< − 2.73) and the survival results. No significant interaction between pathological stage (IVB/I-IVA) and either of LNR (≥0.05/< 0.05)/LODDS (≥ − 2.73/<− 2.73) were observed. In Model 3, pathological stage N1–3 category were significantly shorter DFS (*p* = .02, HR: 3.14, 95% CI: 1.17–8.84) than pathological N0 category. In Model 4, LNR ≥ .05 were significantly shorter MiSGC-specific survival (*p* = .04, HR: 9.01, 95% CI: 1.07–109.7) than LNR < .05. In Model 5, LODDS (≥ − 2.73/< − 2.73) were not associated with survival results.

**Table 3:** Multivariate survival analysis by Cox’s proportional hazards model

Parameter		MiSGC-specific survival	OS	DFS
Model 1				
LNR	HR	7.90	4.84	4.15
(≥0.05/< 0.05)	95% CI	1.54–57.1	1.05–24.8	1.48–11.2
	*p* value	.01	.04	.01
Pathological stage	HR	1.31	1.26	.97
(IVB/I-IVA)	95% CI	0.18–6.73	.17–6.48	.14–4.04
	*p* value	.76	.79	.97
Model 2				
LODDS	HR	3.37	2.34	2.50
(≥ − 2.73/<−2.73)	95% CI	.62–18.3	.46–10.7	.85–6.75
	*p* value	.15	.28	.09
Pathological stage	HR	1.98	1.86	1.37
(IVB/I-IVA)	95% CI	.26–12.0	.24–11.3	.20–6.02
	*p* value	.47	.51	.71
Model 3				
Pathological category	HR	4.21	2.72	3.14
(N1–3/N0)	95% CI	.82–30.5.09	.59–13.9.19	1.17–8.84.02
	*p* value			
Pathological stage	HR	2.81	1.89	1.38
(IVB/I-IVA)	95% CI	.29–10.7	.26–9.78	.21–5.42
	*p* value	.42	.48	.69
Model 4				
LNR	HR	9.01	6.98	2.93
(≥0.05/< 0.05)	95% CI	1.07–109.7	.92–64.1	.84–11.8
	*p* value	.04	.06	.09
Pathological stage	HR	1.34	1.33	.95
(IVB/I-IVA)	95% CI	.18–7.04	.18–7.03	.14–3.97
	*p* value	.75	.75	.95
Vascular invasion	HR	.82	0.59	1.68
(Presence/Absence)	95% CI	.09–9.27	.07–4.87	.41–6.13
	*p* value	.87	.63	.45
Model 5				
LODDS	HR	2.40	1.94	1.77
(≥ − 2.73/<−2.73)	95% CI	.40–14.9	.34–10.5	.57–5.14
	*p* value	.33	.44	.31
Pathological stage	HR	1.66	1.70	1.10
(IVB/I-IVA)	95% CI	.22–10.3	.22–10.6	.16–4.89
	*p* value	.59	.57	.91
Vascular invasion	HR	2.31	1.53	2.51
(Presence/Absence)	95% CI	.38–18.3	.28–8.78	.87–7.79
	*p* value	.36	.62	.09

## Discussion

This study demonstrated that higher LNR in MiSGC significantly predicted shorter MiSGC-specific survival, DFS, and OS in uni−/multi-variate analyses adjusting for the 8th UICC pathological stage.

LNR for survival outcomes in head and neck cancer was a significant predictor in large cohorts and several single institutions [3, 4, 10–12]. Two meta-analyses of 14,254 patients with squamous cell carcinoma (SCC) in oral cavity from 19 articles [10] as well as 4197 patients with laryngeal and hypopharyngeal SCC from 13 articles [11] showed close association between LNR and OS. LNR in our previous studies from a hospital sample was a predictor for OS and disease-specific survival of 46 cases of hypopharyngeal SCC [3], and for OS in 32 major salivary gland carcinomas having various histological classifications [4]. Furthermore, Hong et al. reported in 87 high-grade carcinomas of salivary gland, in whom 95% had a carcinoma in the parotid or submandibular gland, that LNR predicted OS, cancer-specific survival, and DFS [12]. The present results, showing a significant relation between LNR and survival outcomes, agree with previous studies [3, 4, 10–12].

LODDS were recently recognized as a prognosticator of survival results of head and neck cancer (2, 13–15). For oral SCC, LODDS were a predictor of locoregional recurrence [2], and disease-specific survival [13]. For laryngeal SCC, higher LODDS indicated shorter OS and DFS [14]. LODDS in 225 head and neck cancers were associated with shorter OS [15]. The findings of the present study, demonstrating a significant association between LODDS and survival results, are in agreement with those of the previous studies [2, 13–15].

Because pathological stage was possibly a confounding factor in the present study, we examined whether LNR and LODDS predict survival outcomes adjusting with pathological stage. For multivariate Cox’s proportional hazards model, we did not select adjusting factors with *p* < .05 based on univariate Cox’s proportional model or log-rank test. Because pathological stage IVB stage based on pathological T and N category including extranodal extension is comprehensive, we selected adjusting the pathological stage (IVB/I-IVA) for multivariate analysis. Because adjuvant therapy was not pathological factor, we did not select adjusting adjuvant therapy. Similar to the significant results between higher LNR and shorter survival outcomes in uni−/multi-variate analyses of the present study, both LNR and LODDS in laryngeal SCC were predictors of both DFS and OS; LNR whose HR (DFS, 13.49; OS, 10.71) was greater than that of LODDS (DFS, 0.235; OS, 0.287) was a more reliable indicator for evaluating the survival [14]. Considering the significant relation between LNR and survival outcomes in the multivariate analysis of the present and previous studies [14], LNR is considered as an indicator for postoperative radiation or chemoradiation.

As the reason for selecting binary classification in the present study, histological classification (MEC/others) or pathological stage (IVB/I-IVA) was due to MEC with the largest number of patients or pathological stage IVB with comprehensive stage including pathological T category, pathological N category, extranodal extension, respectively.

Because MiSGC in the present study had various histological classification, we considered that pathological stage was not predictive for OS, MiSGC-specific survival, or DFS in either model 1 or 2 in Table 3.

The present study includes certain limitations. Only a small sample size was retrospectively recruited from a single institution because of the rarity of this MiSGC. Therefore, larger cohort from multi-institutions should be prospectively conducted to provide a more precise and useful results from statistical point. A limitation of the present study was no use of a least absolute shrinkage and selectin operator cox proportional hazards regression model to improve the predictive accuracy of survival models in the setting of a relatively small cohort.

## Conclusions

Higher LNR was a significant predictor of shorter MiSGC-specific survival, DFS, and OS in MiSGC.

## Data Availability

The datasets used and/or assessed during this study are available from the corresponding author for reasonable request.

## Abbreviations

LNR: Lymph node ratio
LODDS: Log odds of positive lymph nodes
MiSGC: Minor salivary gland carcinoma
MEC: Mucoepidermoid carcinoma
UICC: Union for International Cancer Control
LRFS: Local recurrence-free survival
RRFS: Regional recurrence-free survival
DMFS: Distant metastasis-free survival
DFS: Disease-free survival
OS: Overall survival
ROC: Receiver operating curve
HR: Hazard ratio
95%CI: 95% confidence interval
SCC: Squamous cell carcinoma
